# Development and Evaluation of New Coupling System for Lower Limb Prostheses with Acoustic Alarm System

**DOI:** 10.1038/srep02270

**Published:** 2013-07-24

**Authors:** Arezoo Eshraghi, Noor Azuan Abu Osman, Hossein Gholizadeh, Jalil Ahmadian, Bizhan Rahmati, Wan Abu Bakar Wan Abas

**Affiliations:** 1 Department of Biomedical Engineering, Faculty of Engineering, University of Malaya, 50603 Kuala Lumpur, Malaysia; 2 Department of Electrical Engineering, Faculty of Engineering, University of Malaya, 50603 Kuala Lumpur, Malaysia; 3 Department of Mechanical Engineering, Faculty of Engineering, University of Malaya, 50603 Kuala Lumpur, Malaysia; 4 Department of Electrical Engineering, Faculty of Engineering, University of Malaya, 50603 Kuala Lumpur, Malaysia; 5 Department of Engineering Design and Manufacture, Faculty of Engineering, University of Malaya, 50603 Kuala Lumpur, Malaysia

## Abstract

Individuals with lower limb amputation need a secure suspension system for their prosthetic devices. A new coupling system was developed that is capable of suspending the prosthesis. The system's safety is ensured through an acoustic alarm system. This article explains how the system works and provides an in vivo evaluation of the device with regard to pistoning during walking. The system was designed to be used with silicone liners and is based on the requirements of prosthetic suspension systems. Mechanical testing was performed using a universal testing machine. The pistoning during walking was measured using a motion analysis system. The new coupling device produced significantly less pistoning compared to a common suspension system (pin/lock). The safety alarm system would buzz if the suspension was going to fail. The new coupling system could securely suspend the prostheses in transtibial amputees and produced less vertical movement than the pin/lock system.

The demand for prosthetic devices, particularly as a result of lower limb amputation, is growing^1^. Amputees face a permanent disability that can only be addressed by artificial limbs (prostheses). As such, advancements in prosthetics technology are of utmost importance to improving the quality of amputees' life. When lower limb prosthesis is in use, the lower limb residuum is required to bear weight, but its soft tissues are not physiologically accustomed to such weight bearing activities^2^,^3^. Therefore, advancements in prosthetics technology are of utmost importance to amputees' life. Lower limb residuum should bear weight while its soft tissues are not physiologically accustomed to weight bearing.

Prosthetic suspension systems play important role in the amputee's ability to perform the various activities associated with everyday life, from quiet standing to intense sport activities^4^. Suspension systems have evolved tremendously to provide more secure ambulation for prosthetic users. Liners are a fundamental element of the suspension system and they not only act as a cushion but also contribute to the overall function of the prosthesis. Roll-on liners, which are mostly made of silicone, are popular due to the fact that they offer improved suspension, cushion and durability^5^. Modern suspension systems are a combination of a roll-on silicone liner and either a pin lock system or a rubber seal. Although these systems have been proven to be successful in suspending above-knee and below-knee prostheses, amputees have reported some difficulties using them^6^,^7^,^8^.

Pin/lock and Seal-In liners are believed to be superior to other suspension methods as they cause the least pistoning (vertical movement) inside the prosthetic socket; this is particularly true of the Seal-In liner^9^,^10^,^11^. Nevertheless, objective and subjective evaluations imply that pin/lock systems are associated with pain and skin problems, particularly at the distal end of the residuum^12^,^13^. Seal-In systems are also said to cause high interface pressure inside the prosthetic socket. Both systems cause discomfort in terms of donning and doffing and this can be particularly challenging for amputees that have poor hand dexterity^9^,^14^.

Bearing in mind the above-mentioned pros and cons, the authors of this paper developed a new coupling system. This paper aims to describe the newly designed system and to evaluate its effectiveness when used for lower limb amputees. The researchers assumed that the new concept of suspension would effectively secure the prostheses to the amputees' residual limbs and have a positive effect on the biomechanics of the prostheses.

## Results

The maximum tensile load that the system could bear was 350.9N (SD 0.5) of tensile loading before the coupling failed. The pin/lock system could tolerate loading of 580.4N (SD 0.1); however, the lock system lost its function after three trials.

**Figure 1:**
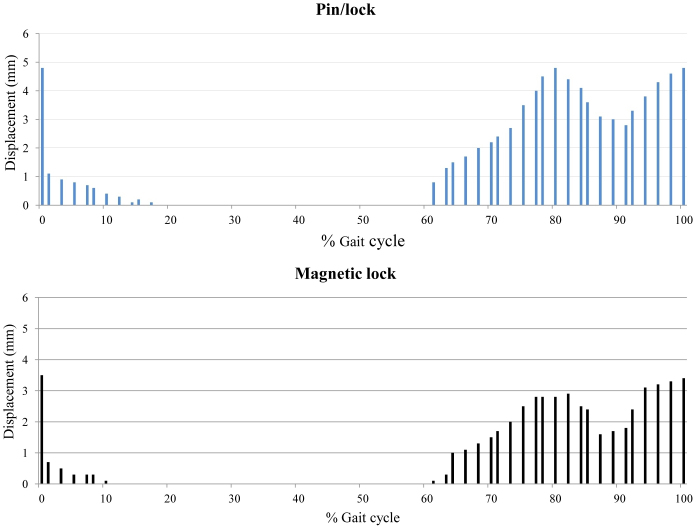
Pistoning measurement. The average pistoning values with the pin/lock and new prosthesis suspension systems during one gait cycle. (n = 13).

The pistoning values for the two systems showed significant differences during the gait cycle. The most significant difference was evident during the swing phase (*P* < 0.05). With the pin/lock, 4.8 mm (SD, 0.3) of pistoning was observed between the liner and the socket at initial contact. This was reduced to 3.5 mm with the new coupling system. From mid stance to initial swing, no pistoning was observed between the liner and socket with any of the systems. Figure 1 shows the pattern of pistoning observed during walking with the two suspension systems.

## Discussion

This article explained the specifications of a new coupling device that could be incorporated in lower limb prostheses. The researchers conjured that the new device was able to retain the prosthesis securely on the amputee's residual limb during ambulation. Mechanical testing showed that the new device could resist tensile loading of up to 350.9N and therefore the prosthetic leg should stand the centripetal force during walking. The force is calculated based on the mass of the prosthetic leg, the distance to the center of mass of the lower leg and the time taken to swing the prosthetic leg during walking^15^. Based on our previous studies, the maximum force that was applied to the prosthetic leg during fast walking was 90N^10^. Therefore, it can be concluded that the system enables successful suspension of prosthesis.

One of the most suitable measures for assessing the effectiveness of suspension is pistoning^4^. Pistoning occurs either between the bone and soft tissue or the skin/liner and socket. This study evaluated the pistoning between the skin-liner and socket wall^4^. The pistoning values during walking with the new device were compatible with that of the pin/lock suspension system. Mainly in the swing phase, the systems demonstrated substantial difference and lower vertical displacement was resulted using the new suspension system. The pistoning was previously evaluated by a static simulation system^9^. However, in this study the newly designed protocol by the authors was adopted to investigate the pistoning during real walking^14^. The results were compared with the previous findings through the simulation protocol^9^. There was not considerable difference between the pistoning values with the two protocols (gait simulation and during real gait).

In healthy individuals, the neurons are responsible for detecting any failure in the body and sending awareness signals to the brain. Users of artificial limbs face many problems as parts of body limbs are missing and prosthetic limbs cannot communicate with the brain to control sensory and motor mechanisms. As such, any failure in the prosthetic components may cause irreparable injuries that can aggravate the challenges that are already present in an amputee's life. Fall risk is high among lower limb amputees, particularly older amputees^16^,^17^; consequently, generating detectable signals at the first moment of failure can reduce fall risk. To the authors' knowledge this is the first prosthesis suspension system that incorporates an alarm system. The coupling alarm system was successfully tested on transtibial amputees. The buzzer can produce a buzz-type alarm for one second. The power supply (1200 mAh 9 V battery) enables the system to be functional up to 2200 hours. The authors are working to improve the alarm system as a wireless connection to the mobile gadgets in order to and show data such as the battery level, and degree of safety by evaluation of the coupling force, and buzzer testing. Furthermore, due to very low power consumption of the new device, it can be equipped with magnetic energy harvesting device to produce required energy. As such, the battery can be simply eliminated from the system that will lead to dramatic system improvement as the user does not need to replace the battery.

The main challenges were to place the sensors so that they could detect the desired signals while ensuring that the system remained user friendly. The sensitivity of the contact sensor was also crucial. If the sensor was too sensitive, a slight vibration during walking could activate the output alarm. This fake alarm could mislead the prosthesis user about the risk of suspension failure.

The new coupling device for the lower limb prosthesis can suspend prosthesis on the amputee's residual limb during walking. The pistoning values observed during walking were comparable to other suspension systems, and in some points of gait cycle were considerably lower. This can be considered the first prosthetic suspension system incorporating a safety alarm system. The alarm system may increase safety level by reducing the risk of fall. Nevertheless, the new coupling system can be both utilized with or without the safety alarm system.

## Methods

### Mechanical coupling device

Prosthetic sockets are important components in prosthesis. Suspension systems are, in effect, coupling devices that are positioned between the prosthetic socket and the distal components of the prosthesis, such as prosthetic foot, ankle and pylon. The main factors that should be taken into account when designing prostheses are durability, cosmetic appearance, comfort, function and cost. With these factors in mind, a 3D mechanical computer-aided design (CAD) program (SolidWorks 2009) was used to accomplish the design. Every lower limb prosthesis for persons with lower limb amputation is consisted of the following components but not limited to: socket, pylon (shank), knee and foot^18^. The prosthetic suspension system is usually positioned either inside the socket or between the prosthetic socket and the pylon. Considering the limited space available at this interface, the dimensions of the coupling system used in this study were designed so that it could fit the socket end of an adult amputee. The limited space also dictated the height of the coupling system so that it could also be used with long residual limbs. The new system was designed to be used with silicone liners as they are widely available and commonly in use. To this end, a cap was designed that matched both the main body of the new coupling device, and the liner's distal end. The dimensions were purposely formulated to match with those of the liner. The cross section was circular and, in order to reduce weight, the cap was hollow. The hollow space incorporated a central screw in the middle and was filled with silicone adhesive to promote firm attachment to the liner. The new coupling idea was based on the magnetic field. As such, the cap was made of mild steel to produce high gripping force.

The body of the coupling device was the source of magnetic power. A permanent Neodymium Iron Boron magnet was utilized that was small but was capable of generating a strong magnetic power. The housing intensified the magnetic field by flanges. In order to control the magnetic power, a mechanical switch was affixed to the housing and the magnet. When the rotary switch was in the “On” position the cap was attracted to the housing, whereas it was released from the lower body of the coupling device when the switch was in the “Off” position.

### Acoustic alarm system

**Figure 2:**
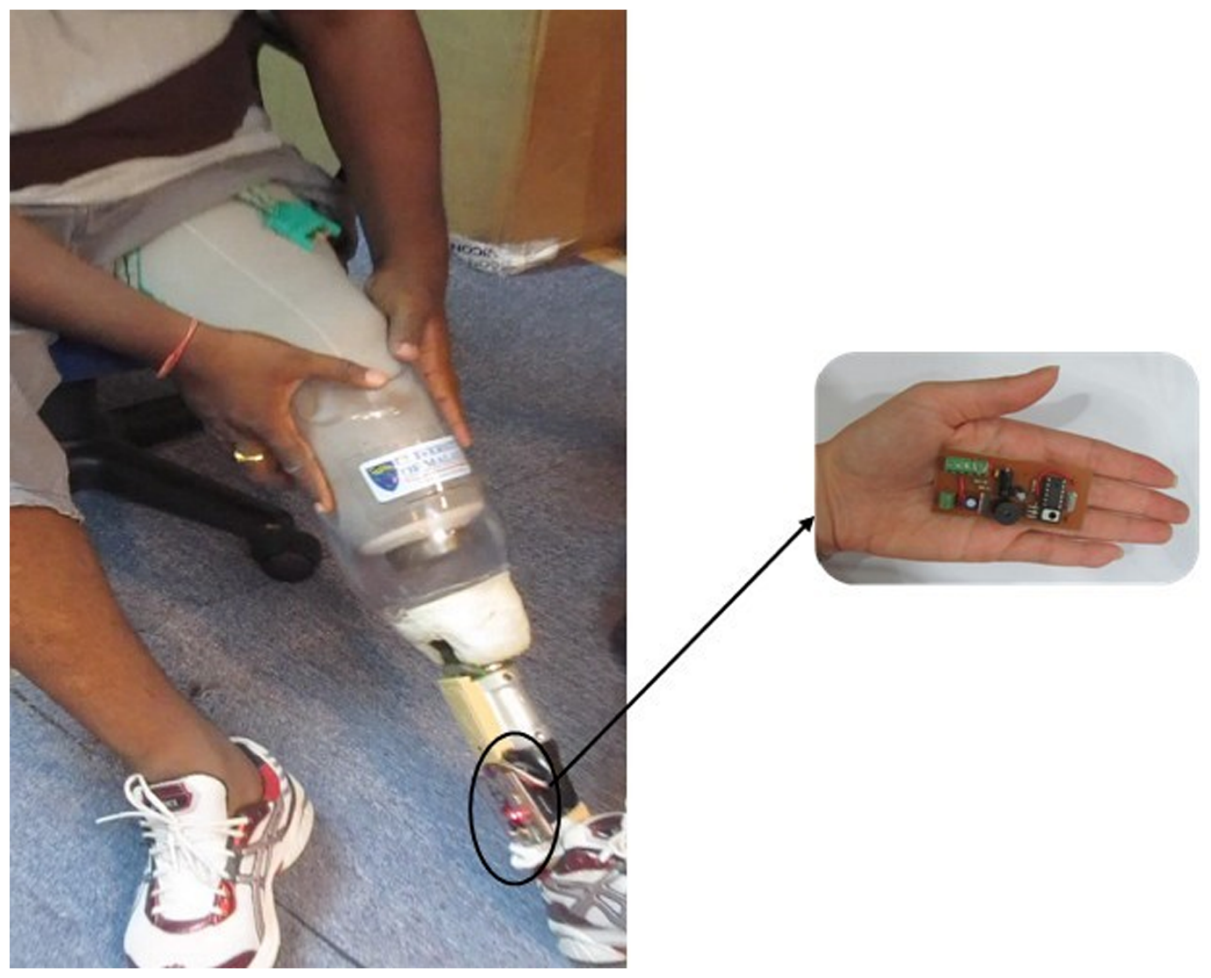
New prosthetic coupling system. A participant is donning a prosthesis that is fitted with the new prosthetic coupling system and the coupling alarm.

**Figure 3:**
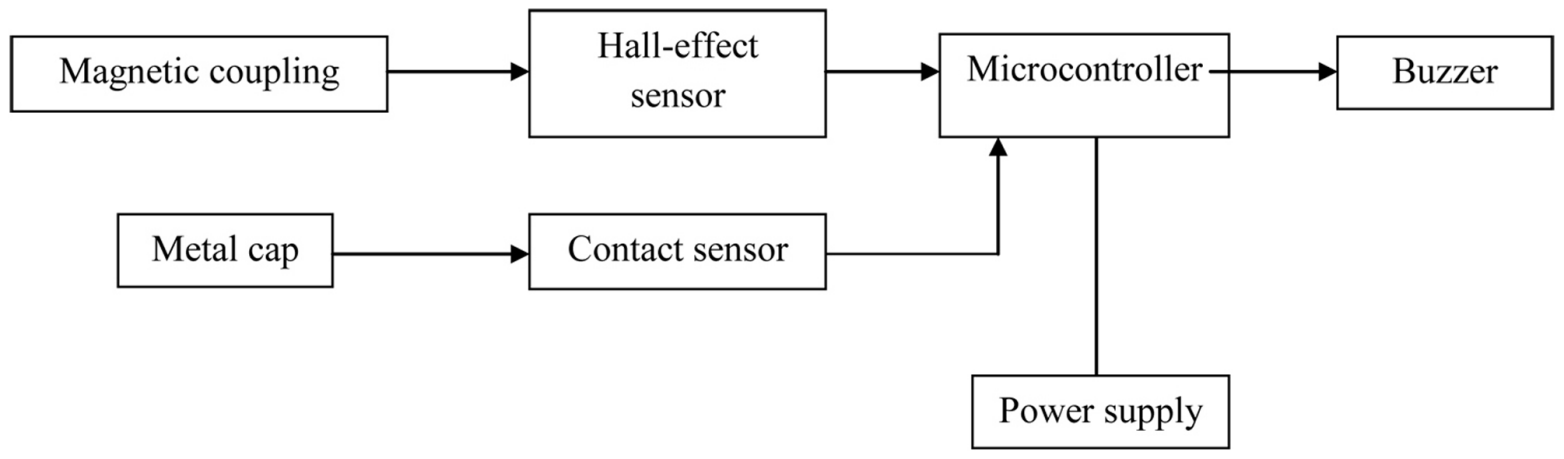
Alarm system. Block diagram of the coupling alarm system.

A coupling alarm system was designed that is capable of detecting any failure in the newly designed coupling system for the lower limb prosthesis. This system consists of an interface, process unit and power supply (Figs. 2, 3). The signals are detected and processed through a micro-controller unit that subsequently makes the appropriate decision as to whether to energize the output or not. The interface consists of two inputs and one output. One hall-effect sensor detects the magnetic field and a contact sensor ensures that the joint remained in total contact with the limb. The output is a buzzer which is energized through a transistor to amplify the microcontroller signals and produce the required alarm. The buzzer produces an audible alarm signal at the level of 97dB with the frequency of 2 KHz.

Power in the range of 2.5 to 5 V was required for the microcontroller. The buzzer and Hall-effect sensor required a 9 V battery. Therefore, a 9-Volt battery, a 1 V regulator and two transistors were utilized. The transistors are responsible for switching the voltage between the microprocessor output and the desired voltage for the Hall-effect sensor and buzzer. The microprocessor is required to distinguish whether the coupling is successfully attained or had failed. If the signals from the Hall-effect sensor show that the magnetic field has activated the coupling, the contact sensor signals are analyzed.

**Figure 4:**
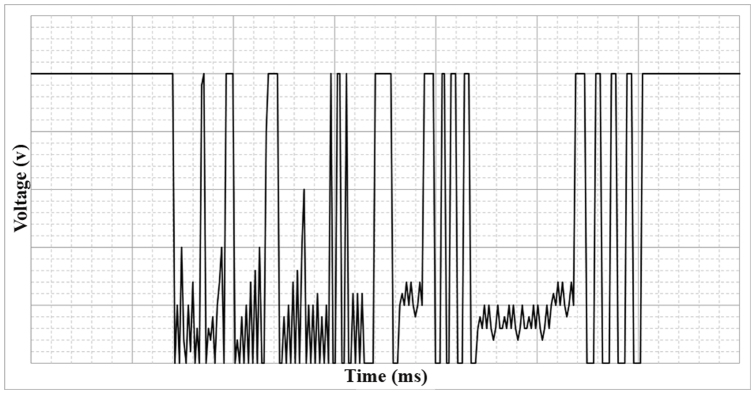
Decision making by the microcontroller. The microprocessor samples data every one millisecond for 3 ms to ensure that the sensor detected the vibration of coupling.

The microcontroller samples every one millisecond for 3 ms. If all data are the same, it would be replaced by the previous. This process is also repeated for three times to ensure that the sensor detected the vibration of coupling; not the detachment (Fig. 4). The final result will be processed by the microprocessor to make appropriate decision. This device is equipped with one 1200 mAh 9 V battery. Energy consumption of different parts is shown in table 1.

### Participants and experiment

**Figure 5:**
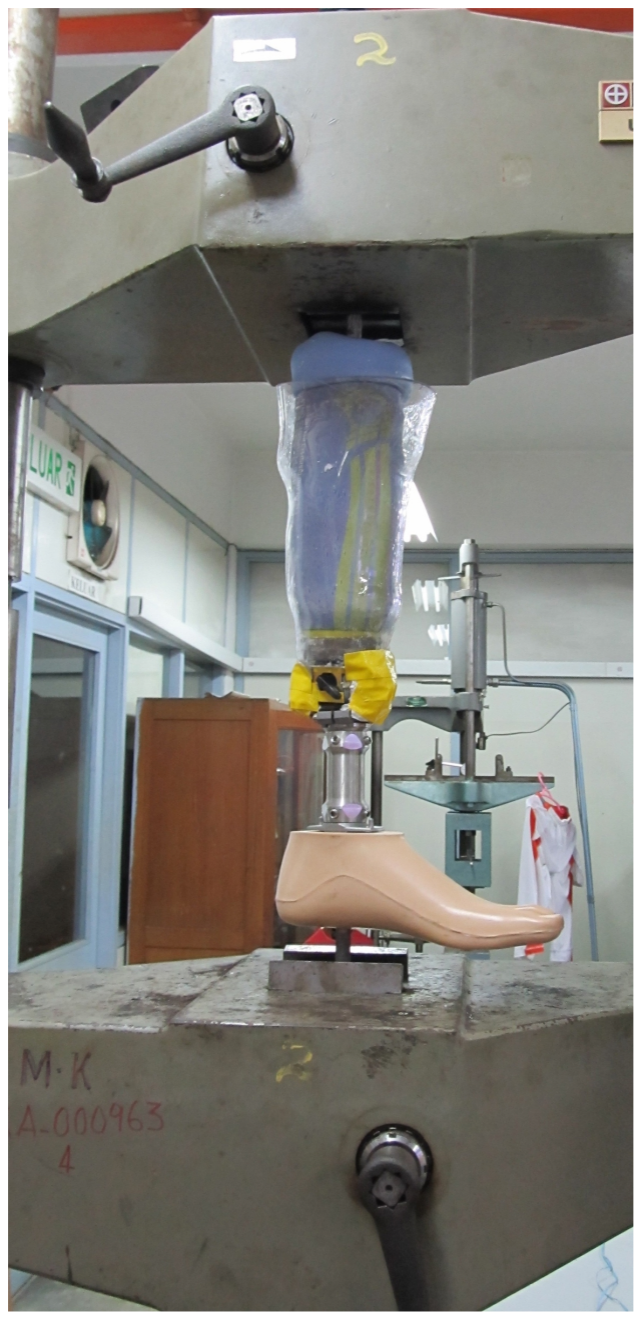
Mechanical testing. Tensile testing for the new prosthesis coupling system.

The study was approved by the Medical Ethics committee, University of Malaya Medical Centre (Reference No. 907.26). Thirteen transtibial amputees participated in the study as sample of convenience. All the participants were using a pin/lock liner with shuttle lock. Following a written informed consent, each participant was provided with a transtibial prosthesis that incorporated the new coupling and alarm system. To ensure consistent prosthetic quality, fabrication and alignment technique, the participants also received one prosthesis with pin/lock coupling system. The participants attended gait training sessions at the Brace and Limb Laboratory, University of Malaya. The suspension system was tested both mechanically and experimentally as the subjects engaged in basic walking activities. Mechanical testing under tensile loading was performed using the universal testing machine INSTRON 4466 through a special jig (Fig. 5). The experimental testing of pistoning during gait was carried out using a 6-camera Vicon motion system. The detailed protocol developed by the authors has been explained elsewhere^14^. However, a brief description is presented here. The Helen Hayes marker set was modified to include 18 reflective markers for the lower limbs. Two extra markers were used specifically to measure the vertical movement or pistoning at the liner-socket interface. The displacement of markers indicated the pistoning. Finally, SPSS 20.0 (SPSS, Chicago, IL) was used to analyze the data through paired-samples t tests with Bonferroni adjustment. The level of significance was set at 0.05.

## Author Contributions

A.E. designed the system and the protocol, fabricated the prostheses, conducted the experiments, collected and analysed the data, discussed the results and drafted the manuscript. N.A.A.O. and W.A.B.W.A. supervised the overall project, and helped in revising the manuscript. H.G. collected and analysed the data, discussed the results, wrote a part of the manuscript and helped in prosthetic fabrication. J.A. designed the system, discussed the results and helped in drafting the manuscript. B.R. conducted and analysed the mechanical testing data and discussed the results. All the authors read and commented on the manuscript.

## Figures and Tables

**Table 1:** The energy consumption of different components of the alarm system

System component	Energy consumption (μA)
Microprocessor unit	15 (in work mode)
	2 (in standby mode)
Magnetic sensor	10
Buzzer	700 (in alarm mode)
Other parts, transistors and regulators	500
